# Plasma Citrulline as a Biomarker in Critical Illness: A Structured Narrative Review

**DOI:** 10.3390/diagnostics16172831

**Published:** 2026-09-02

**Authors:** Oana Frandeș, Alexandra Elena Lazăr, Sergiu Ioan Frandeș, Oana Elena Branea, Leonard Azamfirei

**Affiliations:** 1 Doctoral School of Medicine and Pharmacy, George Emil Palade University of Medicine, Pharmacy, Science and Technology of Targu Mures, 540142 Targu Mures, Romania; oana.frandes@umfst.ro (O.F.); sergiu.frandes@umfst.ro (S.I.F.); 2 Anesthesiology and Intensive Care Department, George Emil Palade University of Medicine, Pharmacy, Science and Technology of Targu Mures, 540142 Targu Mures, Romania; oana.branea@umfst.ro (O.E.B.); leonard.azamfirei@umfst.ro (L.A.)

**Keywords:** plasma citrulline, gastrointestinal dysfunction, biomarker, enterocyte function, critical illness, sepsis, septic shock

## Abstract

**Introduction**: Gastrointestinal dysfunction is common in critically ill patients and is associated with increased morbidity and mortality. Plasma citrulline, a non-proteinogenic amino acid synthesized by enterocytes, has been proposed as a biomarker of intestinal function and enterocyte mass. However, the available evidence regarding its clinical significance in critically ill patients remains heterogeneous. This structured narrative review aimed to evaluate the current evidence on plasma citrulline in critical illness, focusing on its association with gastrointestinal dysfunction, disease severity, and clinical outcomes. **Methods**: A structured narrative review based on a structured literature search was conducted using PubMed and Scopus. Reference lists of included studies and relevant reviews were also screened. Studies evaluating circulating citrulline in adult critically ill patients and reporting gastrointestinal dysfunction, intestinal injury, disease severity, or other relevant clinical outcomes were included. Study selection was performed by two reviewers, and data were analyzed descriptively. **Results**: Eleven studies met the inclusion criteria, the majority being prospective observational studies. The timing of citrulline measurement, patient populations, analytical methods, and evaluated outcomes varied considerably across studies. Lower circulating citrulline concentrations were frequently associated with gastrointestinal dysfunction or markers of intestinal impairment; however, associations with disease severity, mortality, and other clinical outcomes were inconsistent. Several factors, including renal function, systemic inflammation, and methodological variability, may influence the interpretation of circulating citrulline concentrations. **Conclusions**: Plasma citrulline may provide complementary information regarding enterocyte function and gastrointestinal dysfunction in critically ill patients. However, current evidence does not support its use as a standalone diagnostic or prognostic biomarker. Further prospective studies using standardized measurement protocols and serial assessment are needed to determine its potential role alongside clinical evaluation and complementary biomarkers.

## 1. Introduction

Gastrointestinal dysfunction is common in critically ill patients with sepsis and is frequently associated with a poorer prognosis [1,2,3]. Intestinal alterations occurring during sepsis involve multiple mechanisms, including impaired regulation of splanchnic blood flow and intestinal mucosal hypoperfusion, increased intestinal permeability, local tissue perfusion disturbances related to coagulation abnormalities, and changes in the microbiome [4,5,6,7]. In addition, prolonged mechanical ventilation and the use of sedatives may contribute to gastrointestinal pathology by reducing intestinal motility. The gastrointestinal tract can be involved both as the source of sepsis, particularly in intra-abdominal infections, and as a target organ affected during the progression of sepsis and septic shock, contributing to multiple organ dysfunction [4,6].

Citrulline is a non-proteinogenic amino acid synthesized primarily in small bowel enterocytes from glutamine and subsequently metabolized at the renal level [8,9]. Circulating citrulline is considered a biomarker of intestinal epithelial cell mass and function [4,10]. In the kidneys, citrulline serves as a key precursor for de novo arginine synthesis. Arginine is the main substrate of nitric oxide synthase, leading to the production of nitric oxide, an essential mediator of vascular tone, microcirculation, and immune response [11,12].

The diagnosis and assessment of gastrointestinal dysfunction remain challenging for clinicians in intensive care units. Although several severity scoring systems are routinely used in critically ill patients, gastrointestinal dysfunction is not directly represented as a dedicated organ component in the Sequential Organ Failure Assessment (SOFA) score, while global severity scores such as APACHE II and SAPS do not specifically characterize gastrointestinal injury [4,13,14,15]. Unlike other organ systems routinely monitored in the ICU, the gastrointestinal tract lacks validated bedside monitoring tools, and no biomarker has yet demonstrated sufficient accuracy and clinical applicability for routine use [13,16]. Clinical manifestations commonly used in practice, such as feeding intolerance, abdominal distension, vomiting, or high gastric residual volumes, are nonspecific and may not accurately reflect intestinal perfusion, mucosal ischemia, or enterocyte injury [17,18,19]. In addition, the Acute Gastrointestinal Injury (AGI) score is limited by subjective assessment, heterogeneous definitions, and incomplete validation across different ICU populations [13,20]. Consequently, assessment of gastrointestinal dysfunction remains difficult in critically ill patients, contributing to the concept of the gut as an “invisible organ” in intensive care medicine.

Several approaches have been investigated for the assessment of gastrointestinal dysfunction and intestinal perfusion in critically ill patients, including serum lactate, biomarkers of intestinal permeability, gastric ultrasound, and methods of microcirculatory evaluation. However, these techniques also present important limitations. Serum lactate lacks specificity for intestinal injury and may reflect global tissue hypoperfusion rather than isolated gastrointestinal dysfunction. Biomarkers of intestinal permeability and enterocyte injury, such as intestinal fatty acid-binding protein (I-FABP), D-lactate, or lipopolysaccharide, have shown variable results and remain insufficiently validated for routine clinical use [17]. In addition, bedside techniques such as gastric ultrasound may provide indirect information regarding gastric emptying and feeding tolerance but do not directly assess intestinal perfusion or epithelial integrity. Similarly, methods evaluating microcirculation remain technically challenging and are not routinely available in most intensive care settings [21,22]. Taken together, these limitations highlight the need for more reliable biomarkers capable of reflecting enterocyte function and intestinal integrity in critically ill patients.

In this context, plasma citrulline may be useful for the evaluation of gastrointestinal function; however, the published results are heterogeneous. Moreover, the available studies assess different outcomes, further complicating the evaluation of citrulline as a biomarker in clinical practice. The interpretation of plasma citrulline levels is also complicated by multiple confounding factors, including renal function, systemic inflammation, nutritional status, and timing of measurement [1,8,23,24].

This review aims to synthesize the current evidence on plasma citrulline in critically ill patients by examining its association with gastrointestinal dysfunction, disease severity, and clinical outcomes, as well as the limitations that currently limit clinical use.

## 2. Methods

This structured narrative review was based on a structured literature search. Although the review was not designed as a systematic review, selected elements of the PRISMA reporting framework were used to improve the transparency of study identification and selection.

A literature search was conducted in PubMed and Scopus to identify studies evaluating circulating citrulline in critically ill adult patients. The reference lists of included studies and relevant reviews were also manually screened.

The search strategy combined terms related to citrulline, critical illness, and gastrointestinal dysfunction or intestinal injury. Outcome-related terms, such as mortality or disease severity, were not required in the search strategy in order to avoid excluding studies evaluating gastrointestinal dysfunction without explicitly reporting these terms.

The PubMed search included combinations of MeSH terms and free-text keywords related to citrulline, critical illness, and gastrointestinal dysfunction or intestinal injury. The Scopus search used the corresponding terms within the TITLE-ABS-KEY field. No publication date restrictions were applied. The search was limited to articles published in English. The complete search strategies used for PubMed and Scopus are provided in Supplementary Table S1. The final search was conducted on 20 August 2026 and identified 41 records in PubMed and 76 records in Scopus.

Screening and study selection were performed by two reviewers (O.F. and S.I.F.). Records were screened based on titles and abstracts, and potentially relevant articles were subsequently assessed in full text. Disagreements regarding study eligibility were resolved through discussion.

Studies were eligible for inclusion if they included adult critically ill or ICU patients, evaluated circulating citrulline as a biomarker, and reported gastrointestinal dysfunction, intestinal injury, disease severity, or other relevant clinical outcomes. Studies involving pediatric or non-ICU populations, experimental or animal studies, studies without circulating citrulline measurements, interventional studies not evaluating citrulline as a biomarker, and studies without relevant clinical outcome data were excluded. Studies evaluating urinary citrulline were considered only when circulating citrulline was also measured and relevant to the study objectives.

**Figure 1:**
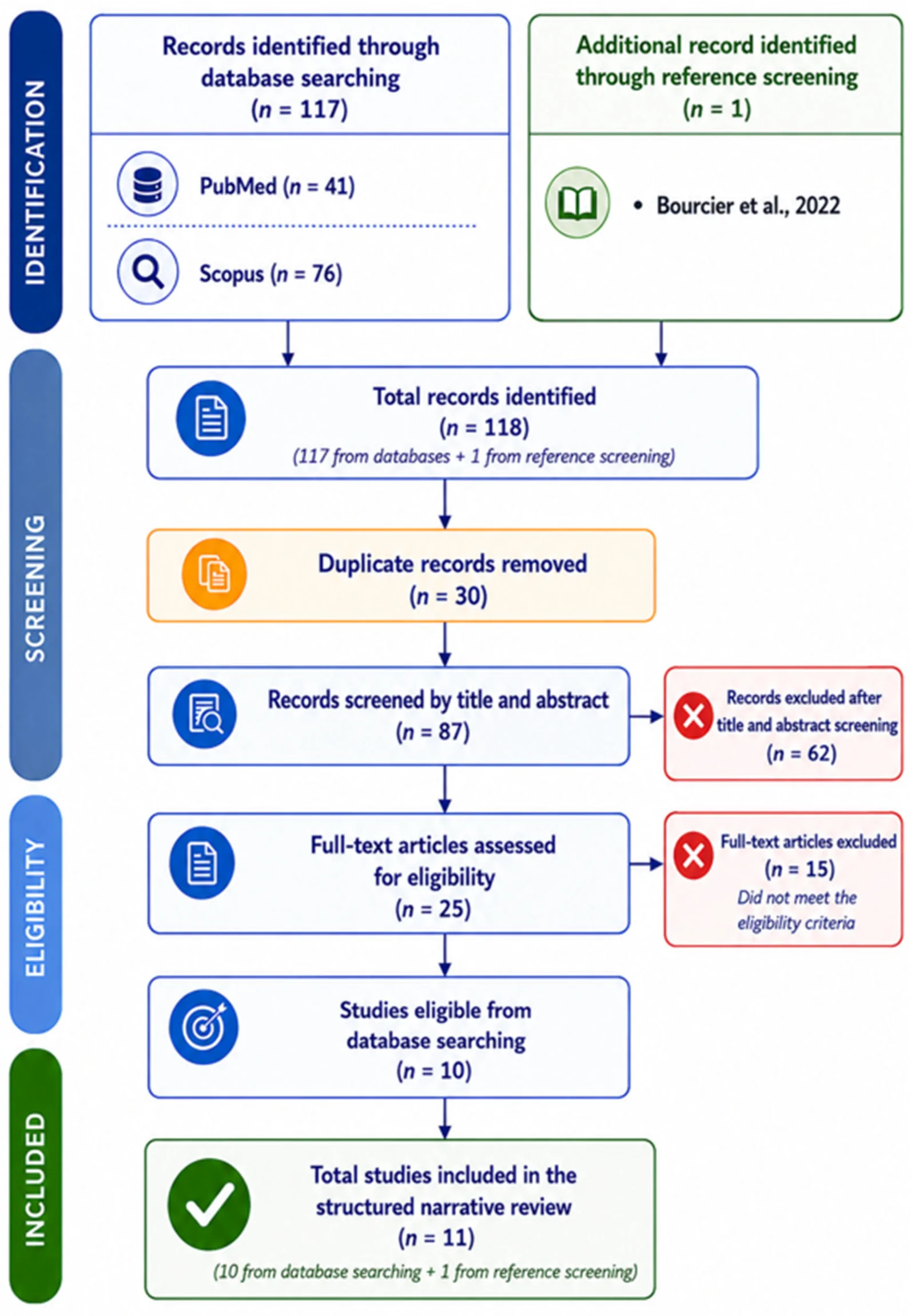
Simplified flow diagram of study identification and selection [25].

Data extracted from the included studies included study design, patient population, sample size, citrulline measurement method, timing of sampling, evaluated gastrointestinal parameters, clinical outcomes, and the main findings. Given the heterogeneity in patient populations, study designs, timing of citrulline measurement, analytical methods, and reported outcomes, the findings were synthesized narratively. The study selection process is summarized in Figure 1.

Overall, 11 studies were included in the final analysis.

Artificial intelligence-assisted tools (ChatGPT GPT-5.6 Luna), OpenAI) were used during the preparation of this manuscript for the generation of the figures and language editing. The generated content was reviewed and edited by the authors, who take full responsibility for the final version of the manuscript.

## 3. Results

### 3.1. Characteristics of Included Studies

A total of 11 studies were included in this structured narrative review. Most were prospective observational studies, although the study populations and clinical settings were heterogeneous. Sample sizes ranged from 16 to 151 participants. The included studies evaluated critically ill adults with different clinical conditions, including general ICU populations, sepsis or septic shock, multiple organ dysfunction, acute respiratory distress syndrome, gastrointestinal dysfunction, and suspected non-occlusive mesenteric ischemia.

Plasma or serum citrulline was measured at different time points, ranging from a single measurement at ICU admission to serial assessments during the first days of critical illness. Considerable methodological heterogeneity was also observed, with different analytical techniques used for citrulline determination, including ion-exchange chromatography, HPLC, tandem mass spectrometry, LC-MS/MS, and ELISA. This analytical heterogeneity should also be considered when comparing absolute citrulline concentrations across studies, as measurements obtained using ELISA may not be directly interchangeable with those obtained by chromatographic or mass spectrometry-based methods. The clinical outcomes were also heterogeneous and included gastrointestinal dysfunction or failure, organ dysfunction, intestinal ischemia, ARDS, bacterial translocation, and mortality. The main characteristics of the included studies are summarized in Table 1.

### 3.2. Citrulline Levels in Critical Illness

Most studies evaluating gastrointestinal dysfunction reported lower plasma citrulline concentrations in patients with impaired intestinal function. Noordally et al. found that only patients with intestinal dysfunction had plasma citrulline concentrations below 15 µmol/L, while no significant associations were observed with CRP, renal failure, SOFA score, or APACHE II score [1]. Similarly, Fagoni et al. reported lower citrulline concentrations in patients with gastrointestinal dysfunction or failure [28]. Teng et al. and Tyszko et al. also reported lower citrulline concentrations in patients with more severe gastrointestinal dysfunction, supporting an association between reduced citrulline levels and the severity of intestinal impairment [4,29].

Poole et al. reported significantly lower plasma citrulline concentrations in critically ill patients than in healthy controls, while Tyszko et al. found lower citrulline concentrations in patients with sepsis compared with controls [4,27].

However, this association was not consistent across all studies. In the prospective cohort study by Onuk et al., serum citrulline concentrations were not associated with gastrointestinal dysfunction assessed using the GIDS and showed no correlation with gastric ultrasound parameters [30]. Similarly, Bourcier et al. found limited diagnostic value of plasma citrulline for identifying non-occlusive mesenteric ischemia or intestinal necrosis in critically ill patients [25]. In the Onuk study, neither serum citrulline nor I-FABP correlated with gastrointestinal dysfunction, whereas gastric antral cross-sectional area showed better discrimination. Together, these findings highlight the limited diagnostic performance of citrulline when used as an isolated biomarker in specific clinical settings [25,30].

The main findings of the included studies are summarized in Table 2.

### 3.3. Association with Disease Severity and Outcomes

Several studies investigated the relationship between plasma citrulline concentrations and disease severity or clinical outcomes; however, the evaluated outcomes differed considerably across studies.

Piton et al. demonstrated that low plasma citrulline concentrations were independently associated with increased 28-day mortality. In the first study, a plasma citrulline concentration ≤10 µmol/L measured 24 h after ICU admission independently predicted mortality, while a subsequent study identified a threshold of ≤12.2 µmol/L as the optimal prognostic cut-off [23,24].

Other studies focused primarily on gastrointestinal severity rather than mortality. Fagoni et al. reported significantly lower plasma citrulline concentrations in patients with gastrointestinal dysfunction or failure than in patients without gastrointestinal impairment [28]. Similarly, Teng et al. demonstrated a progressive decrease in plasma citrulline concentrations with increasing severity of gastrointestinal dysfunction and reported significant correlations with both APACHE II and SOFA scores [29]. Tyszko et al. also observed lower plasma citrulline concentrations in patients with septic shock compared with sepsis and in patients with more severe acute gastrointestinal injury, suggesting that plasma citrulline reflects gastrointestinal dysfunction severity [4].

In contrast, not all studies found an association between plasma citrulline concentrations and mortality. Crenn et al. reported a marked decline in plasma citrulline concentrations during the early phase of septic shock but found no relationship with mortality [8]. Similarly, Tyszko et al. observed no significant differences in plasma citrulline concentrations between survivors and non-survivors despite lower concentrations in patients with more severe gastrointestinal dysfunction [4].

### 3.4. Temporal Aspects of Citrulline Measurement

The timing of plasma citrulline measurement varied considerably across the included studies. Most studies performed a single determination, typically at ICU admission or within the first 24 h [1,4,26,28]. However, several studies included serial measurements at different time points, ranging from the first hours after admission to multiple days during ICU stay [4,8,23,29].

Some studies evaluated citrulline only at admission, while others performed serial measurements and observed dynamic changes during the course of critical illness. For example, Piton et al. reported a significant decrease in plasma citrulline concentrations during the first 24 h after ICU admission, whereas Crenn et al. demonstrated a rapid decline following the onset of septic shock [8,23]. Similarly, Tyszko et al. and Teng et al. monitored plasma citrulline over several days, showing that changes in citrulline concentrations paralleled the evolution of gastrointestinal dysfunction [4,29].

These observations suggest that serial assessment of plasma citrulline may provide additional clinically relevant information compared with a single measurement. However, the available evidence remains limited, as studies used different sampling schedules, evaluated different clinical outcomes, and included relatively small patient populations. Consequently, current data are insufficient to determine the optimal timing or frequency of plasma citrulline measurements in critically ill patients.

### 3.5. Factors Influencing Plasma Citrulline Concentrations

Renal function was approached from two different perspectives in the included studies. Some studies excluded patients with chronic kidney disease [23,24,29], while the study published by Noordally et al. investigated the association between plasma citrulline levels and renal function but did not find a significant association [1]. One study indirectly evaluated renal function by comparing patients requiring continuous renal replacement therapy (CRRT) with those who did not require CRRT, without identifying significant differences in plasma citrulline levels [4]. However, these findings should be interpreted cautiously, as renal function represents an important component of citrulline metabolism. Reduced renal uptake and conversion of citrulline to arginine may alter circulating concentrations, while acute kidney injury, renal replacement therapy, and the overall severity of critical illness may further complicate this relationship.

The relationship between systemic inflammation and plasma citrulline was evaluated in only a limited number of studies. Piton et al. reported an inverse correlation between plasma citrulline concentrations and CRP, whereas Crenn et al. observed a similar association with CRP but found no significant relationship with TNF-α or IL-10 [8,23].

Nutritional status and mode of feeding were evaluated in one study, which compared patients receiving enteral nutrition, parenteral nutrition, both, or no nutritional support. The study did not find a significant association between plasma citrulline levels and mode of nutritional support or nutritional markers such as albumin and prealbumin [1].

## 4. Limitations of Current Evidence

Heterogeneity was observed across multiple domains, including study design, patient population, measurement techniques, and outcome definitions.

Most of the included studies were observational, predominantly prospective, and conducted in single-center settings. Sample sizes were generally small, and study populations were heterogeneous, including patients with sepsis, septic shock, and varying degrees of organ dysfunction. These factors limit the generalizability of the findings.

The assessment of plasma citrulline levels varied across studies, with differences in analytical methods. Most studies used chromatographic methods, while others used different techniques such as LC-MS/MS or ELISA. In addition, the timing of measurement was inconsistent, ranging from single determinations at ICU admission to serial measurements over several days. These represent major limitations for the interpretation of citrulline levels in both clinical and predictive contexts.

Several potential confounding factors were variably addressed across studies. Renal function was handled inconsistently across studies. Some investigators excluded patients with severe renal dysfunction, whereas others evaluated its influence without identifying a significant association with plasma citrulline concentrations. However, because renal conversion of citrulline to arginine represents a key step in citrulline metabolism, impaired kidney function remains an important biological confounder that may influence circulating citrulline concentrations independently of intestinal function.

Finally, none of the included studies evaluated whether citrulline-guided assessment or monitoring could improve clinical decision-making or patient outcomes. Therefore, although low circulating citrulline concentrations were frequently associated with gastrointestinal dysfunction or markers of disease severity, the current evidence does not establish how citrulline measurements should be incorporated into routine clinical practice.

## 5. Discussion

### 5.1. Main Findings

This review indicates that reduced circulating citrulline concentrations are frequently observed in critically ill patients and are commonly associated with gastrointestinal dysfunction or markers of intestinal impairment. However, the strength and clinical relevance of these associations vary across patient populations and clinical settings. While several studies reported lower citrulline concentrations in patients with more severe gastrointestinal dysfunction or adverse clinical outcomes [1,8,23,24,28,29], these findings support the role of citrulline as a potential marker of enterocyte mass and intestinal impairment in this population.

However, the relationship between citrulline levels and clinically relevant outcomes remains inconsistent. While some studies reported an association between low citrulline levels and increased mortality or disease severity [4,23,24,29], others did not confirm these findings [1,8], suggesting that citrulline alone may not be a reliable prognostic marker.

Taken together, the available evidence supports a strong biological rationale for citrulline as a marker related to enterocyte function, but does not support its use as a standalone diagnostic or prognostic biomarker in critically ill patients. Its association with gastrointestinal dysfunction appears more consistent than its association with mortality or overall disease severity.

### 5.2. Interpretation of Inconsistencies

Although lower plasma citrulline concentrations were frequently observed in patients with gastrointestinal dysfunction, their association with mortality and other clinical outcomes was less consistent. These discrepancies probably reflect the complex biology of plasma citrulline rather than a simple lack of biomarker performance [4,8,23,28,29].

Unlike organ-specific biomarkers, plasma citrulline reflects only one component of gastrointestinal dysfunction, namely enterocyte functional mass [9,17,21,31]. Gastrointestinal dysfunction during critical illness is a multifactorial process involving impaired microcirculation, intestinal hypoperfusion, epithelial barrier disruption, altered motility, systemic inflammation, and metabolic disturbances. Consequently, plasma citrulline should be interpreted as an adjunctive biomarker rather than a standalone indicator of gastrointestinal injury [14,22].

Furthermore, the different clinical outcomes evaluated across the included studies may also explain the inconsistent findings. While some investigations assessed mortality, others focused on gastrointestinal dysfunction, ARDS, glucose absorption, or organ failure, making direct comparison difficult and highlighting the need for outcome-specific validation [4,23,26,27,28,29].

This distinction may partly explain the inconsistent findings observed across the included studies. The negative results reported by Onuk et al. and Bourcier et al. illustrate that circulating citrulline may have limited diagnostic performance when used as an isolated biomarker in specific clinical settings [25,30]. Similarly, studies evaluating different outcomes—including gastrointestinal dysfunction, intestinal ischemia, ARDS, impaired absorption, organ failure, and mortality—addressed biologically distinct processes. Therefore, an association between citrulline and one clinical outcome should not be assumed to translate directly into predictive value for another.

### 5.3. Pathophysiological Meaning

Plasma citrulline is primarily synthesized by enterocytes of the small intestine from glutamine and plays a central role in the intestinal–renal axis of arginine metabolism. It is subsequently converted to arginine in the kidneys, providing a key substrate for nitric oxide production, which is essential for vascular tone regulation, microcirculation, and immune function [8,10,11].

In critically ill patients, several pathophysiological mechanisms may contribute to decreased plasma citrulline levels. Intestinal hypoperfusion and mucosal ischemia, which are common in sepsis and septic shock, can lead to enterocyte injury and reduced citrulline synthesis [4,10,32]. In addition, systemic inflammation may further impair intestinal function and amino acid metabolism, contributing to lower circulating citrulline levels, although the relationship between citrulline and inflammatory markers has been inconsistent, with some studies reporting correlations with markers such as CRP, but not with others including TNF-α or IL-10 [1,9].

Septic microcirculatory dysfunction and heterogeneous blood flow distribution may impair mucosal oxygen delivery despite apparently preserved systemic hemodynamics [33,34,35,36]. Reduced oxygen delivery to enterocytes may compromise their metabolic activity and decrease plasma citrulline synthesis. Endothelial dysfunction, altered capillary permeability, and septic shunting further contribute to intestinal barrier disruption and enterocyte dysfunction [37,38]. These alterations may reduce functional enterocyte mass and consequently contribute to lower circulating plasma citrulline concentrations. Moreover, mitochondrial dysfunction and impaired cellular oxygen utilization, often described in sepsis-related “cytopathic hypoxia,” may contribute to altered enterocyte metabolism and persistent organ dysfunction despite adequate macrocirculatory resuscitation [39,40,41]. These mechanisms suggest that plasma citrulline does not simply reflect intestinal perfusion but rather the functional integrity and metabolic activity of enterocytes, both of which may be impaired during critical illness.

These mechanisms support the hypothesis that plasma citrulline reflects, at least in part, enterocyte mass, intestinal functional integrity, and the impact of microcirculatory and metabolic disturbances occurring during critical illness. In addition, a study published by Elkhatib et al. supports this hypothesis by presenting citrulline as a biomarker of enterocyte function in inflammatory bowel disease [31]. However, it is not a specific marker, as its levels may also be influenced by extra-intestinal factors such as renal metabolism and systemic inflammatory response.

Furthermore, gastrointestinal dysfunction associated with multiple organ dysfunction syndrome (MODS) is thought to be closely related to intestinal hypoperfusion and ischemia–reperfusion injury. These mechanisms may contribute to disruption of the intestinal mucosal barrier and activation of immunoinflammatory pathways by releasing biologically active mediators into both the systemic circulation and the mesenteric lymphatics [42,43].

However, circulating citrulline is not a specific marker of intestinal dysfunction, as its concentration may also be influenced by extra-intestinal factors, particularly renal metabolism and systemic inflammation. The kidney plays a central role in the uptake and conversion of citrulline to arginine; therefore, impaired renal function may alter circulating citrulline concentrations independently of intestinal enterocyte function. In critically ill patients, the effects of acute kidney injury, renal replacement therapy, altered arginine metabolism, and systemic inflammation may further complicate the interpretation of a single citrulline measurement.

**Figure 2:**
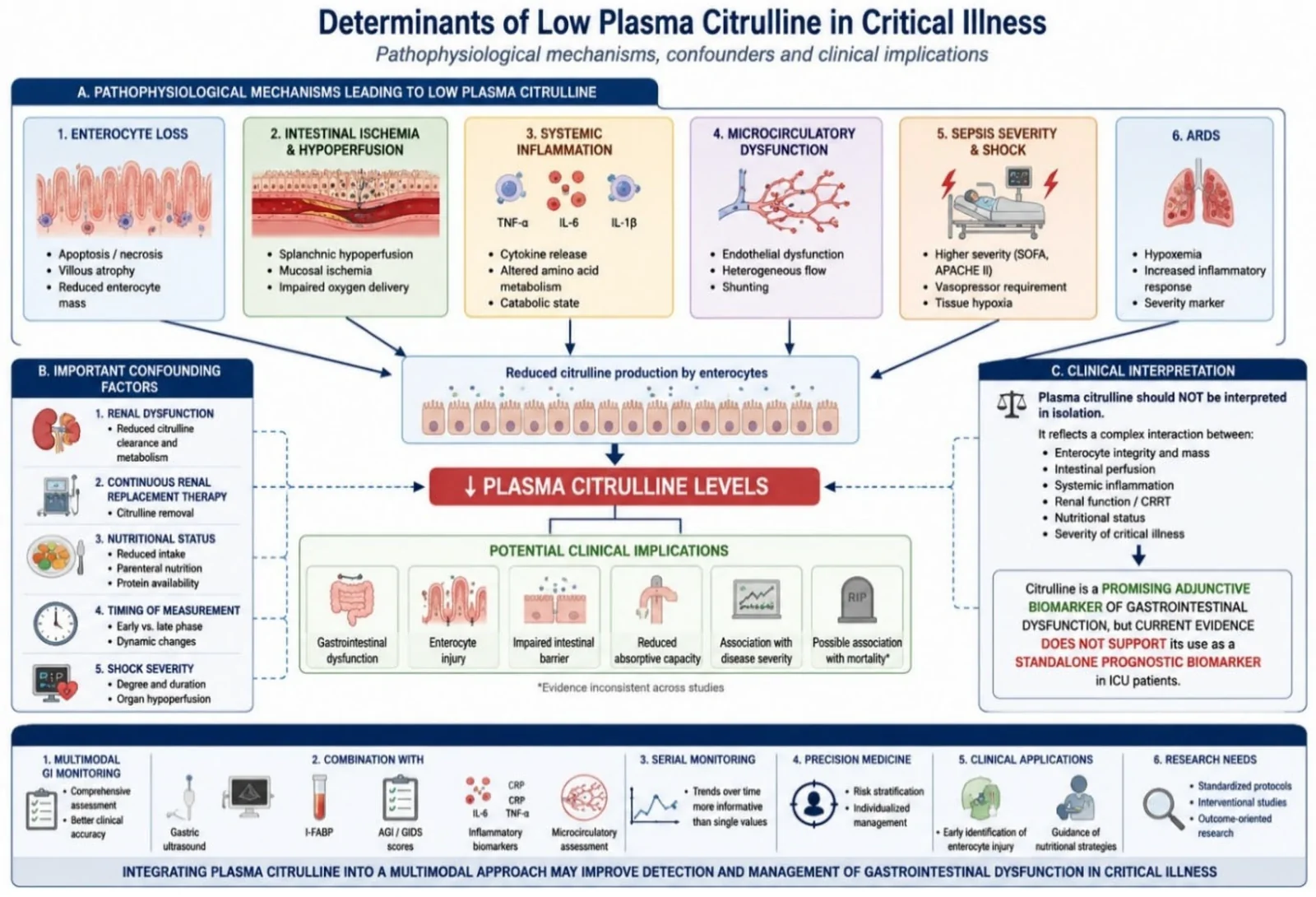
Conceptual overview of factors potentially associated with reduced plasma citrulline concentrations in critical illness. The figure summarizes proposed pathophysiological mechanisms, observed associations, and potential confounding factors. Proposed clinical applications should be considered hypothetical and require prospective validation.

Therefore, a low circulating citrulline concentration should be interpreted as a potential indicator of impaired enterocyte function within a broader clinical and biological context rather than as a direct or isolated measure of gastrointestinal dysfunction. The potential mechanisms contributing to reduced plasma citrulline levels in critically ill patients, as well as major confounding factors and possible clinical implications, are summarized in Figure 2.

### 5.4. Clinical Relevance

Although plasma citrulline is closely linked to intestinal function, its clinical utility in critically ill patients remains limited. The available studies are heterogeneous and show inconsistent associations with clinical outcomes, making it difficult to use citrulline as a reliable marker for prognosis or risk stratification in routine practice [44,45].

Rather than representing a comprehensive marker of gastrointestinal dysfunction, plasma citrulline may reflect one component of intestinal pathophysiology, particularly enterocyte functional mass and metabolic activity. Its interpretation should therefore be integrated with the clinical assessment of gastrointestinal function and, where appropriate, with complementary measures reflecting other aspects of intestinal injury or dysfunction. However, whether such an approach improves diagnostic accuracy or clinical decision-making has not yet been established [9,44,45].

The clinical implementation of plasma citrulline has likely been limited by the heterogeneity of available studies, the absence of standardized cutoff values, variability in analytical methods, and the influence of multiple confounding factors such as renal dysfunction and systemic inflammation. Plasma citrulline levels may not exclusively reflect intestinal injury, as they are also influenced by renal metabolism. Under physiological conditions, citrulline is converted to arginine in the kidneys, whereas acute kidney injury may alter this process by reducing renal conversion and modifying circulating citrulline concentrations independently of enterocyte function [45]. Consequently, renal dysfunction represents an important biological confounder that should be considered when interpreting plasma citrulline levels. Although several included studies did not identify a significant association between renal function and plasma citrulline concentrations, most were relatively small and were not specifically designed to evaluate this relationship. Therefore, the absence of a statistically significant association should not be interpreted as evidence that renal function has no influence on plasma citrulline metabolism.

Although plasma citrulline has a strong biological basis as a marker of enterocyte function, it cannot currently be recommended as a routine biomarker for gastrointestinal dysfunction in critically ill patients. Several factors limit its clinical use. No validated diagnostic or prognostic cutoff has been established, and the optimal timing of measurement remains uncertain. In addition, different analytical methods are used across studies, plasma citrulline is not routinely available as a rapid bedside test, and its interpretation may be influenced by important confounding factors such as renal dysfunction and systemic inflammation. Moreover, although lower plasma citrulline levels have been associated with gastrointestinal dysfunction and, in some studies, with adverse outcomes, there is currently no evidence that their measurement changes clinical management or improves patient outcomes [44,45,46]. Therefore, plasma citrulline should currently be considered an adjunctive biomarker rather than a routine clinical tool.

### 5.5. Comparison with Other Intestinal Biomarkers

Plasma citrulline reflects only one aspect of intestinal pathophysiology and should be distinguished from biomarkers that reflect direct enterocyte injury, epithelial barrier disruption, or microbial translocation. Among the most extensively studied complementary biomarkers, intestinal fatty acid-binding protein (I-FABP) appears particularly relevant. While reduced citrulline concentrations are generally considered to reflect impaired enterocyte functional mass or metabolic activity, increased I-FABP concentrations reflect enterocyte injury and cellular damage. Therefore, these biomarkers may provide complementary rather than interchangeable information [4,47,48].

This distinction is supported by studies evaluating both biomarkers in critically ill patients. Tyszko et al. observed significant alterations in both citrulline and I-FABP during sepsis and septic shock, although their prognostic performance for mortality was limited. In contrast, the AGI score showed stronger prognostic value, suggesting that biomarker measurements cannot replace clinical assessment of gastrointestinal dysfunction [4]. Similarly, Onuk et al. found that neither serum citrulline nor I-FABP was associated with gastrointestinal dysfunction assessed using the GIDS, whereas gastric ultrasound parameters demonstrated better discrimination [30]. These findings further support the need to distinguish between biochemical evidence of enterocyte dysfunction or injury and the broader clinical manifestations of gastrointestinal dysfunction.

Other biomarkers have been proposed to reflect additional components of intestinal barrier dysfunction. Tight-junction-related proteins and markers such as zonulin, claudins, or occludin may provide information related to epithelial barrier integrity, whereas markers associated with bacterial translocation or systemic consequences of barrier dysfunction may reflect different downstream processes. However, evidence supporting their clinical utility in critically ill patients remains limited, and substantial methodological heterogeneity exists regarding assay selection, timing of measurement, and outcome definitions [49].

Rather than suggesting that a specific biomarker panel is already ready for clinical use, the current evidence supports a multidimensional approach for future research. Citrulline could be evaluated alongside a marker of enterocyte injury, such as I-FABP, and interpreted together with standardized clinical assessment of gastrointestinal dysfunction. Additional variables reflecting systemic disease severity and important confounders, including renal function and inflammatory status, may further improve the interpretation of circulating citrulline. Whether such a multimarker approach improves diagnostic accuracy or patient outcomes, however, remains to be established [50].

Compared with these biomarkers, plasma citrulline provides complementary information by reflecting enterocyte functional mass rather than intestinal permeability or epithelial injury alone [4,31]. However, plasma citrulline is also influenced by important biological confounders, including renal function and systemic inflammation. Consequently, no single biomarker currently provides a comprehensive assessment of gastrointestinal dysfunction in critically ill patients. A multimodal approach integrating plasma citrulline with clinical evaluation, severity scores, and other intestinal biomarkers may therefore represent a more appropriate strategy for future clinical practice.

### 5.6. Future Directions

Further research is needed to better define the role of plasma citrulline in critically ill patients. Future studies should include larger and more homogeneous patient populations in order to improve comparability between results. Standardization of citrulline measurement, including analytical methods, cutoff values, and timing of assessment, is also necessary to reduce variability across studies. In particular, longitudinal studies with serial measurements may provide better insight into the dynamic evolution of gastrointestinal dysfunction during critical illness compared to single time-point determinations.

An appropriate prospective study design could include critically ill adults with gastrointestinal dysfunction and a comparison group of critically ill patients without gastrointestinal dysfunction. Plasma citrulline could be measured at ICU admission and at predefined intervals during the first days of critical illness, together with standardized gastrointestinal dysfunction scores, markers of enterocyte injury such as I-FABP, and variables reflecting renal function, systemic inflammation, and overall disease severity. Relevant endpoints could include the development or progression of gastrointestinal dysfunction, gastrointestinal failure, and clinically relevant outcomes such as ICU length of stay and mortality.

Given the complex and multifactorial nature of gastrointestinal dysfunction in critically ill patients, plasma citrulline is unlikely to provide sufficient information as a standalone biomarker. Future approaches should therefore evaluate multimodal gastrointestinal assessment strategies integrating citrulline with standardized clinical scores such as AGI or GIDS, complementary biomarkers reflecting enterocyte injury, and other measures of gastrointestinal function, including gastric ultrasound or markers of intestinal perfusion.

Recent studies combining plasma citrulline with ultrasound-based assessment of gastrointestinal function suggest that multimodal evaluation may improve the assessment of gastrointestinal dysfunction in critically ill patients. However, citrulline alone showed limited predictive performance in this context [30].

Moreover, interventional studies are required to evaluate whether citrulline can be used to guide clinical decisions or therapeutic strategies, particularly in the context of gastrointestinal dysfunction. In this context, emerging evidence suggests that L-citrulline supplementation may increase plasma L-arginine levels more effectively than direct arginine administration, as it bypasses hepatic metabolism [51]. However, clinical data remain limited, and a recent study in mechanically ventilated ICU patients without sepsis did not demonstrate a significant improvement in organ dysfunction or clinical outcomes following enteral citrulline supplementation [46]. These findings highlight the need for further well-designed interventional studies in this area.

### 5.7. Limitations of This Review

This review has several limitations. Although a structured and transparent literature search was performed, the review was not designed as a systematic review, and no formal risk-of-bias assessment was conducted. Therefore, the findings should be interpreted as a structured narrative synthesis rather than as a comprehensive systematic evaluation of the available evidence. In addition, despite expanding the search beyond PubMed, the possibility that relevant studies were missed cannot be excluded. The relatively small number of included studies, their clinical and methodological heterogeneity, and the limited availability of adjusted effect estimates further restricted direct comparison and quantitative synthesis. Finally, publication bias and selective reporting cannot be excluded.

The potential clinical applications illustrated in Figure 2 should be interpreted as hypotheses for future investigation rather than established benefits or validated clinical applications.

## 6. Conclusions

Plasma citrulline concentrations are frequently reduced in critically ill patients and are often associated with gastrointestinal dysfunction. However, their relationship with disease severity and mortality remains inconsistent, and current evidence does not support plasma citrulline as a standalone diagnostic or prognostic biomarker. Future studies should use standardized measurement protocols and evaluate citrulline serially alongside clinical assessment and complementary biomarkers to determine its potential role in multimodal gastrointestinal monitoring.

## Figures and Tables

**Table 1:** Characteristics of studies included in the review.

Author (Year)	Country	Study Design	Population	Comparators/Groups Analyzed	Sample Size (*n*)	Citrulline Measurement Timing	Measurement Method	Main Outcome
Piton et al. (2010) [23]	France	Prospective, observational	Critically ill patients with expected ICU stay >48 h	Shock vs. non-shock; survivors vs. non-survivors	67	Admission, 12 h, 24 h, and day 7	Ion-exchange chromatography (Hitachi L-8800)	28-day mortality
Noordally et al. (2012) [1]	Belgium	Prospective, observational	Critically ill patients with expected ICU stay >48 h and a nasogastric tube	Intestinal dysfunction vs. no intestinal dysfunction	91	At ICU admission	HPLC (Hitachi)	Intestinal dysfunction, SOFA, APACHE II, mortality
Piton et al. (2013) [24]	France	Prospective, observational	Critically ill patients with expected ICU stay >48 h	Shock vs. non-shock; survivors vs. non-survivors	103	Admission and 24 h	Ion-exchange chromatography (Hitachi L-8800)	SOFA, 28-day mortality
Ware et al. (2013) [26]	USA	Observational	Critically ill patients with severe sepsis	ARDS vs. no ARDS	135	At admission	Amino acid analyzer (Hitachi L-8800)	Development of ARDS
Crenn et al. (2014) [8]	France	Prospective, observational	Critically ill patients with septic shock and MODS	Survivors vs. non-survivors; with vs. without bacterial translocation	16	6 h, 12 h, 18 h, 24 h, and day 6	Ion-exchange chromatography (Hitachi L-8800)	Bacterial translocation, inflammatory markers, mortality
Poole et al. (2015) [27]	Austria	Prospective, observational	Mechanically ventilated critically ill patients (>72 h)	Critically ill patients vs. healthy controls	35 (20 critically ill patients; 15 controls)	Baseline and serial measurements over 4 h	Tandem mass spectrometry (API 4000 AB SCIEX)	Relationship between glucose absorption and small intestinal function
Fagoni et al. (2020) [28]	Italy	Prospective, observational	Critically ill patients with expected ICU stay >48 h	With vs. without GID/GIF; with vs. without MOF	39	ICU admission in patients with GID/GIF; before ICU discharge in patients without GID/GIF	Ion-exchange chromatography (Biochrom 30+)	Gastrointestinal dysfunction/failure and MOF
Teng et al. (2021) [29]	China	Retrospective, observational	Critically ill patients with gastrointestinal dysfunction/failure	GID (AGI II) vs. GIF (AGI III–IV) vs. healthy controls	151 (110 critically ill patients; 41 controls)	Within 24 h of ICU admission and after 7 days of treatment	LC-MS/MS	Gastrointestinal dysfunction/failure severity, GIF score, APACHE II
Bourcier et al. (2022) [25]	France	Prospective, multicenter observational	Critically ill patients with suspected non-occlusive mesenteric ischemia	Confirmed NOMI vs. ruled-out NOMI; intestinal necrosis vs. no intestinal necrosis	61	At study inclusion	Plasma amino acid measurement	Diagnosis of NOMI, intestinal necrosis, and prognosis
Onuk et al. (2023) [30]	Turkey	Prospective cohort	Critically ill patients receiving enteral nutrition and remaining in the ICU ≥48 h	GI dysfunction vs. no GI dysfunction	39	Baseline and days 3 and 5 of enteral nutrition	Serum citrulline measurement	GI dysfunction assessed using GIDS; comparison with I-FABP and gastric ultrasound
Tyszko et al. (2023) [4]	Poland	Prospective, observational	Critically ill patients with sepsis or septic shock	Sepsis vs. septic shock vs. controls without infection	68 (58 patients; 10 controls)	Days 1, 3, 5, 7, and 10	ELISA (Cusabio Biotech)	Gastrointestinal dysfunction

AGI, acute gastrointestinal injury; ARDS, acute respiratory distress syndrome; GIDS, Gastrointestinal Dysfunction Score; GIF, gastrointestinal failure; ICU, intensive care unit; I-FABP, intestinal fatty acid-binding protein; NOMI, non-occlusive mesenteric ischemia; OR, odds ratio; SOFA, Sequential Organ Failure Assessment.

**Table 2:** Reported plasma citrulline concentrations and principal clinical findings in the included studies.

Study	Outcome/Comparison	Main Findings	Effect Estimate or Diagnostic Performance	Adjusted Analysis/Relevant Findings
Piton et al. (2010) [23]	28-day mortality	Plasma citrulline decreased during the first 24 h. Lower concentrations were associated with increased 28-day mortality.	Citrulline ≤10 µmol/L at 24 h was associated with mortality.	Independent association with 28-day mortality: OR 8.70; SOFA ≥ 8: OR 15.08.
Noordally et al. (2012) [1]	Intestinal dysfunction	Patients with intestinal dysfunction were the only group with plasma citrulline <15 µmol/L. No association was found with SOFA, APACHE II, CRP, renal failure, or survival.	Citrulline <15 µmol/L associated with intestinal dysfunction (*p* = 0.014).	No independent multivariable estimate reported.
Piton et al. (2013) [24]	28-day mortality	Lower citrulline concentrations at ICU admission were associated with increased mortality.	Citrulline ≤12.2 µmol/L was associated with 28-day mortality.	Independent association: OR 5.17 (95% CI 1.59–16.86).
Ware et al. (2013) [26]	ARDS in severe sepsis	Citrulline was significantly lower in patients with ARDS. Mortality was not associated with citrulline among patients with ARDS.	ARDS: 6.0 vs. 10.1 µmol/L; *p* = 0.002. Lowest quartile: ARDS incidence 50% vs. 15% in highest quartile.	OR for ARDS per 5 µmol/L decrease: 1.50 (95% CI 1.14–1.98); association remained significant after adjustment for severity and shock.
Crenn et al. (2014) [8]	Bacterial translocation and mortality	Lower citrulline concentrations were associated with bacterial translocation and systemic inflammation. Mortality association was not consistently demonstrated.	Association with bacterial translocation reported.	No robust independent prognostic estimate reported.
Poole et al. (2015) [27]	Small intestinal glucose absorption	Critically ill patients had lower citrulline and lower glucose absorption than healthy controls, but citrulline was not significantly correlated with glucose absorption.	Citrulline: 15.2 vs. 26.5 µmol/L; *p* < 0.01. Correlation with glucose absorption: r = 0.28, *p* = 0.12.	No independent analysis reported.
Fagoni et al. (2020) [28]	Gastrointestinal dysfunction/failure and multiple organ failure	Lower plasma citrulline concentrations were observed in patients with gastrointestinal dysfunction/failure and were associated with multiple organ failure.	Lower concentrations in patients with more severe gastrointestinal dysfunction.	Association mainly reported as group differences; no validated cutoff for clinical use.
Teng et al. (2021) [29]	Severity of gastrointestinal dysfunction/failure	Citrulline concentrations were lower in patients with gastrointestinal failure than in those with less severe dysfunction and were associated with GIF score and APACHE II.	Lower concentrations in more severe AGI/GIF categories.	Mainly correlation/group comparison analyses.
Bourcier et al. (2022) [25]	NOMI and intestinal necrosis	Plasma citrulline showed limited diagnostic value for distinguishing NOMI or intestinal necrosis.	No clinically useful diagnostic discrimination was demonstrated.	In contrast, I-FABP showed better diagnostic performance for intestinal necrosis.
Onuk et al. (2023) [30]	Gastrointestinal dysfunction assessed using GIDS	Serum citrulline was not correlated with GI dysfunction or gastric ultrasound parameters and showed poor predictive value.	No significant association with GI dysfunction (*p* > 0.05).	No independent association reported.
Tyszko et al. (2023) [4]	Gastrointestinal dysfunction and AGI severity	Lower citrulline concentrations were associated with more severe gastrointestinal dysfunction, although associations with mortality were inconsistent.	Serial changes in citrulline according to AGI severity were reported.	No validated prognostic cutoff.

## Data Availability

No new data were created or analyzed in this study. Data sharing is not applicable to this article.

## Supplementary Materials

The following supporting information can be downloaded at: https://www.mdpi.com/article/10.3390/diagnostics16172831/s1, Table S1: The complete search strategies used for PubMed and Scopus.
