# Supplementary material for: Plasma Citrulline as a Biomarker in Critical Illness: A Structured Narrative Review
Source: Diagnostics (Basel). 2026 Sep 2;16(17):2831. doi: 10.3390/diagnostics16172831 (PMC13565053; doi:10.3390/diagnostics16172831)
Supplement: Supplementary file 1 [file diagnostics-16-02831-s001.zip › diagnostics-4483032-supplementary.pdf]

Table S1. The complete search strategies used for PubMed and Scopus.

Database	Search strategy	Date of last search	Records identified
PubMed	("Citrulline"[MeSH Terms] OR citrulline[Title/Abstract] OR "L-citrulline"[Title/Abstract] OR "plasma citrulline"[Title/Abstract] OR "serum citrulline"[Title/Abstract]) AND ("Critical Illness"[MeSH Terms] OR "Sepsis"[MeSH Terms] OR "critical illness"[Title/Abstract] OR "critically ill"[Title/Abstract] OR sepsis[Title/Abstract] OR "septic shock"[Title/Abstract] OR ICU[Title/Abstract] OR "intensive care"[Title/Abstract]) AND ("Gastrointestinal Dysfunction"[Title/Abstract] OR "gastrointestinal injury"[Title/Abstract] OR "intestinal injury"[Title/Abstract] OR "intestinal dysfunction"[Title/Abstract] OR "intestinal ischemia"[Title/Abstract] OR "mesenteric ischemia"[Title/Abstract] OR "intestinal failure"[Title/Abstract] OR "enterocyte injury"[Title/Abstract] OR "feeding intolerance"[Title/Abstract] OR AGI[Title/Abstract] OR GIDS[Title/Abstract])	20 August 2026	41
Scopus	TITLE-ABS-KEY(citrulline OR "L-citrulline" OR "plasma citrulline" OR "serum citrulline") AND TITLE-ABS-KEY("critical illness" OR "critically ill" OR sepsis OR "septic shock" OR ICU OR "intensive care" OR "intensive care unit") AND TITLE-ABS-KEY("gastrointestinal dysfunction" OR "gastrointestinal injury" OR "acute gastrointestinal injury" OR "intestinal injury" OR "intestinal dysfunction" OR "intestinal ischemia" OR "mesenteric ischemia" OR "intestinal failure" OR "enterocyte injury" OR "intestinal permeability" OR "gut dysfunction" OR "feeding intolerance" OR AGI OR GIDS)	20 August 2026	76
